# Current Smoking Does Not Modify the Treatment Effect of Intravenous Thrombolysis in Acute Ischemic Stroke Patients—A *Post-hoc* Analysis of the WAKE-UP Trial

**DOI:** 10.3389/fneur.2019.01239

**Published:** 2019-11-22

**Authors:** Ludwig Schlemm, Anna Kufner, Florent Boutitie, Alexander Heinrich Nave, Christian Gerloff, Götz Thomalla, Claus Z. Simonsen, Ian Ford, Robin Lemmens, Keith W. Muir, Norbert Nighoghossian, Salvador Pedraza, Martin Ebinger, Matthias Endres

**Affiliations:** ^1^Klinik und Hochschulambulanz für Neurologie, Charité–Universitätsmedizin Berlin, Berlin, Germany; ^2^Center for Stroke Research Berlin (CSB), Charité–Universitätsmedizin, Berlin, Germany; ^3^Berlin Institute of Health (BIH), Berlin, Germany; ^4^Department of Neurology, Jüdisches Krankenhaus, Berlin, Germany; ^5^Service de Biostatistique, Hospices Civils de Lyon, Lyon, France; ^6^Laboratoire de Biométrie et Biologie Evolutive, Equipe Biostatistique-Santé, Centre National de la Recherche Scientifique, UMR 5558, Villeurbanne, France; ^7^DZHK (German Center for Cardiovascular Research), Partner Site, Berlin, Germany; ^8^Klinik und Poliklinik für Neurologie, Kopf- und Neurozentrum, Universitätsklinikum Hamburg–Eppendorf, Hamburg, Germany; ^9^Department of Neurology, Aarhus University Hospital, Aarhus, Denmark; ^10^Robertson Centre for Biostatistics, University of Glasgow, Glasgow, United Kingdom; ^11^Department of Neurology, University Hospitals Leuven, Leuven, Belgium; ^12^Department of Neurosciences, Experimental Neurology, KU Leuven–University of Leuven, Leuven, Belgium; ^13^Laboratory of Neurobiology, VIB-KU Leuven Center for Brain Disease Research, Leuven, Belgium; ^14^Institute of Neuroscience and Psychology, University of Glasgow, Glasgow, United Kingdom; ^15^Department of Stroke Medicine, Université Claude Bernard Lyon 1, Lyon, France; ^16^Department of Stroke Medicine, Hospices Civils de Lyon, Lyon, France; ^17^Department of Radiology, Hospital Universitari Doctor Josep Trueta, Institut d'Investigació Biomèdica de Girona, Girona, Spain; ^18^Department of Neurology, Medical Park Berlin Humboldtmühle, Berlin, Germany; ^19^DZNE (German Center for Neurodegenerative Diseases), Partner Site, Berlin, Germany

**Keywords:** ischemic stroke, thrombolysis (tPA), acute therapy, smoking, treatment outcome and efficacy, cerebrovascular diseases

## Abstract

**Background:** The “smoking paradox” indicates that patients with acute ischemic stroke (AIS) who smoke at the time of their stroke may have a better prognosis after intravenous thrombolysis than non-smokers. However, findings are inconsistent and data analyzing the effect of smoking on treatment efficacy of intravenous thrombolysis are scarce.

**Methods:** We performed a pre-specified *post-hoc* subgroup analysis of the Efficacy and Safety of MRI-Based Thrombolysis in Wake-Up Stroke (WAKE-UP) trial that randomized AIS patients with unknown time of symptom onset who had diffusion-weighted imaging-fluid attenuation inversion recovery (DWI-FLAIR) mismatch to either alteplase or placebo. Patients were categorized as current smokers or non-smokers (including former smokers and never-smokers). Baseline demographic and clinical characteristics, as well as clinical and imaging follow-up data were analyzed according to smoking status.

**Results:** Four hundred and eighty six patients were included in the analysis. Current smokers (133, 27.4%) were younger (60.1 ± 13.0 vs. 67.2 ± 10.3 years; *p* < 0.001) and less often had arterial hypertension (45.0% vs. 56.8%; *p* = 0.02) or atrial fibrillation (3.8% vs. 15.3%; *p* < 0.001). The acute stroke presentation was more often due to large vessel occlusion among current smokers (27.1 vs. 16.2%; *p* = 0.01), and smokers had a trend towards more severe strokes (National Institutes of Health Stroke Scale score>10 in 27.1% vs. 19.5%; *p* = 0.08). The treatment effect of alteplase, quantified as odds ratio for a favorable outcome (modified Rankin Scale [mRS] score at 90 days of 0 or 1), did not differ between current smokers and non-smokers (*p*-value for interaction: 0.59). After adjustment for age and stroke severity, neither the proportion of patients with favorable outcome, nor the median mRS score at 90 days differed between current smokers and non-smokers. When additional potential confounders were included in the model, the median mRS score was higher in current smokers than in non-smokers (cOR of better outcome for current smokers vs. non-smokers: 0.664 [0.451–0.978], *p* = 0.04).

**Conclusions:** In patients with mild to moderate MRI-proven AIS and unknown time of symptom onset with DWI-FLAIR mismatch, current smokers had worse functional outcome as compared to non-smokers. Current smoking did not modify the treatment effect of alteplase.

**Clinical Trial registration:** Main trial (WAKE-UP): ClinicalTrials.gov, NCT01525290; and EudraCT, 2011-005906-32. Registered 02 February 2012.

## Introduction

Intravenous thrombolysis with recombinant tissue plasminogen activator (alteplase) is an effective treatment option for eligible patients with acute ischemic stroke (AIS) that improves functional outcome (1, 2) and reduces long-term disability (3). Knowledge of the risk/benefit profile of intravenous thrombolysis in specific patient groups may aid in individualizing treatment decisions. There is conflicting evidence in the literature as to whether current smoking is associated with a better prognosis in patients with AIS treated with alteplase (4–9). More importantly, there are no data that describe the potential effect of current smoking as a treatment effect modifier of intravenous thrombolysis with alteplase in patients with MRI-proven AIS. In the NINDS t-PA stroke trial, a subgroup analysis of which indicated that there was no interaction between current smoking and treatment effect of alteplase (10), AIS was defined clinically without confirmatory MRI (11). It has been hypothesized that current smoking may modify the treatment effect of alteplase by lowering levels of endogenous tissue plasminogen activator. This would induce a hypercoagulable state which in turn favors the formation of fibrin-rich thrombi that are more susceptible to fibrinolytic treatment (12).

In the current study, we examined whether smoking status at the time of stroke occurrence modified the treatment effect of alteplase in terms of functional recovery in patients with AIS enrolled in the Efficacy and Safety of MRI-Based Thrombolysis in Wake-Up Stroke (WAKE-UP) trial (13).

## Materials and Methods

### Participants and Trial Design

WAKE-UP was a multi-center, two-arms, interventional, prospective, double blind, randomized controlled trial that compared the effect of treatment with alteplase and placebo in patients with AIS and unknown time of symptom onset in whom magnetic resonance imaging (MRI) suggested that the stroke had occurred within a time window of 4.5 h. The detailed protocol and the main results have been published previously (13). Patients aged 18–80 years old were enrolled and randomized in a 1:1 ratio to treatment with either alteplase at a dose of 0.9 mg/kg body weight or placebo if they had no known contraindications (except unknown time of symptom onset), were independent in their daily living before the stroke incident, were not planned to receive mechanical thrombectomy, and exhibited a diffusion-weighted imaging-fluid attenuated inversion recovery (DWI-FLAIR) mismatch on acute MRI. Patients were recruited 70 experienced stroke research centers in Europe (13). Randomization occurred by means of a Web-based procedure with a permuted-block design according to trial center. During the ascertainment of clinical and baseline characteristics, patients were asked about smoking status. Response options included “current smoker” (regular tobacco use within the last 12 months), “ex-smoker” (no tobacco use within the last 12 months), and “never-smoker.” For all analyses, never-smokers and ex-smokers were combined into a single group of (current) non-smokers to increase statistical power.

### Outcome Measures

The main outcome measure in our analysis was the modification of the treatment effect of alteplase as compared to placebo associated with smoking status. The treatment effect of alteplase was quantified as the odds ratio (OR) of a favorable outcome at 90 days as determined by a score of 0 or 1 on the modified Rankin scale (mRS, scale ranging from 0 to 6, with higher scores reflecting higher degree of disability), or as the common OR of improved functional outcome in an ordinal mRS shift analysis. Secondary outcome measures were functional outcome at 90 days and the occurrence of hemorrhagic complications or death stratified by smoking status.

### Statistical Analysis

Continuous variables are presented as mean ± standard deviation (SD) or as median and interquartile range (IQR). Categorical data are presented as counts and percentages. Between-group differences were tested for statistical significance in both univariate analyses and multivariate analyses adjusted for age and baseline stroke symptom severity (National Institutes of Health Stroke Scale [NIHSS] score) using chi-squared tests, Student's *t*-tests, Wilcoxon–Mann–Whitney U test, and binary and cumulative logit models. The treatment effect of alteplase was expressed as an OR or common OR with 95% confidence intervals and calculated using an unconditional logistic-regression model or a proportional-odds logistic-regression, respectively, adjusted for the two randomization strata (age and baseline NIHSS score, pre-specified). Large vessel occlusion included occlusion of the internal carotid artery; middle, anterior, and posterior cerebral artery; basilar artery; and vertebral artery and was determined based on acute magnetic resonance angiography by the central reading board. Modification of the treatment effect by smoking status was assessed by looking at the interactive term. A two-sided alpha-level of 0.05 was considered statistically significant. As a *post-hoc* addition to the analysis plan, we repeated the multivariate analyses including factors that were distributed unevenly between the two groups (*p* < 0.05) as additional covariates, namely age as a continuous variable, the presence of proximal large vessel occlusion at baseline, medical history of atrial fibrillation, and medical history of arterial hypertension. In addition, we performed exploratory analyses of the effect of ever-smoking (combining current smokers and ex-smokers) vs. never-smoking (never-smokers) on outcome and treatment effect of alteplase.

### Ethics Approval and Informed Consent

The WAKE-UP trial protocol was approved by the national regulatory authority in each participating country. The trial was approved by the respective national or local ethics committees or institutional review boards. Patients or their legal representatives provided written informed consent according to national and local regulations. The subgroup analysis with regard to smoking status presented in this paper was approved by the trial steering committee. The data that support the findings of this study are available from Dr. Thomalla (thomalla@uke.de) upon reasonable request.

## Results

### Patient Characteristics at Baseline

Information on smoking status was available for 486 of 503 patients (96.6%), of whom 230 (47.3%) were classified as never-smokers, 123 (25.3%) as ex-smokers, and 133 (27.4%) as current smokers. Demographic and clinical characteristics of all patients at baseline according to smoking status are shown in Table 1. Patients in the current-smokers group were, on average, younger (mean age 60.1 ± 13.0 vs. 67.2 ± 10.3; *p* < 0.001) and less often had a medical history of arterial hypertension (45.0 vs. 56.8%; *p* = 0.02) or atrial fibrillation (3.8 vs. 15.3%; *p* < 0.001). At the acute stroke presentation, current smokers more frequently had large vessel occlusion than non-smokers (27.1 vs. 16.2%; *p* = 0.01). In addition, there was a trend for current-smokers to be more likely to present with more severe strokes (NIHSS score >10; 27.1 vs. 19.5%; *p* = 0.08), although median NIHSS scores did not differ. The remaining parameters, including imaging findings and procedural time intervals, were similar between the groups of current smokers and non-smokers.

**Table 1:** Demographic and clinical characteristics at baseline according to smoking status.

**Variable**	**Non-smokers (*n* = 353)**	**Current smokers (*n* = 133)**	*P*-value^*^
Mean age ± SD—years	67.2 ± 10.3	60.1 ± 13.0	<0.001
Male sex—no./total no. (%)	221/353 (62.6)	91/133 (68.4)	0.24
**Reason for unknown time of symptom onset—no./total no. (%)**			
Nighttime sleep	320/353 (90.7)	115/133 (86.5)	0.35
Daytime sleep	13/353 (3.7)	10/133 (7.5)	
Aphasia, confusion, or other	20/353 (5.7)	7/133 (5.3)	
Median interval between last time the patient was known to be well and symptom recognition (IQR)—hours	7.3 (4.8–8.9)	7.0 (4.8 – 8.8)	0.63
**Medical history—no./total no. (%)**			
Arterial hypertension	200/352 (56.8)	59/131 (45.0)	0.02
Diabetes mellitus	56/349 (16.0)	25/132 (18.9)	0.78
Hypercholesterolemia	128/337 (38.0)	50/130 (38.5)	0.57
Atrial fibrillation	53/346 (15.3)	5/132 (3.8)	<0.001
History of ischemic stroke	45/352 (12.8)	22/133 (16.5)	0.49
Median NIHSS score (IQR)	5 (4–9)	6 (4–11)	0.34
**NIHSS score, stratified—no./total no. (%)**			
≤10	284/353 (80.5)	97/133 (72.9)	0.08
>10	69/353 (19.5)	36/133 (27.1)	
Median lesion volume on diffusion-weighted imaging (IQR)—ml	2.2 (07–7.5)	2.2 (0.9–10.0)	0.33
Any large vessel occlusion on time-of-flight magnetic resonance angiography—no. (%)^†^	55/340 (16.2)	35/129 (27.1)	0.01
Median time from symptom recognition to treatment initiation (IQR)—hr	3.2 (2.5–3.9)	3.0 (2.5–3.7)	0.34
Interval between last time that the patient was last known to be well and treatment initiation (IQR)—hr	10.5 (8.1–12.1)	10.1 (8.1–12.1)	0.51

* P-value not adjusted for multiple testing.

† *Includes occlusion of the internal carotid artery; middle, anterior, and posterior cerebral artery; basilar artery; and vertebral artery. SD stands for standard deviation; IQR, interquartile range; NIHSS, National Institutes of Health Stroke Scale; MRI, magnetic resonance imaging*.

### Prognostic Value of Current Smoking Status—Follow-Up Data

**Figure 1:**
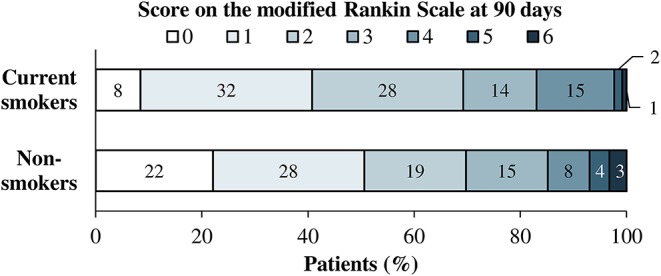
Distribution of scores on the modified Rankin scale at 90 days according to smoking status.

In univariate analyses, fewer patients in the group of current smokers than in the group of non-smokers had a favorable outcome (mRS score 0–1), but this difference did not reach statistical significance (40.8 vs. 50.6%; *p* = 0.06). Similarly, the median mRS score at day 90 tended to be higher in current smokers than in non-smokers (2 [1–3] vs. 1 [1–3]). The distribution of mRS scores at 90 days according to smoking status is shown in Figure 1. After adjustment for age and initial NIHSS score, there was no statistically significant difference between current smokers and non-smokers with regards to the proportion of favorable outcome at 90 days (OR for current smokers vs. non-smokers: 0.728 [0.462–1.148], *p* = 0.13) or the distribution of mRS scores at 90 days (mRS shift analysis; common OR of better outcome for current smokers vs. non-smokers: 0.828 [0.569–1.203], *p* = 0.32). When age taken as a continuous variable, the presence of proximal large vessel occlusion at baseline and medical history of atrial fibrillation and arterial hypertension were included as additional covariates in the regression model, the difference in the distribution of mRS scores at 90 days between current smokers and non-smokers reached statistical significance (mRS shift analysis; common OR of better outcome for current smokers vs. non-smokers: 0.664 [0.451–0.978], *p* = 0.04). No significant between-group differences were observed for deaths at 90 days or for signs of intracranial hemorrhage, symptomatic hemorrhage, new ischemic lesions, and space occupying infarctions as detected by MRI (Table 2).

**Table 2:** Safety outcomes according to smoking status.

**Variable**	**Non-smokers (*n* = 353)**	**Current smokers (*n* = 133)**	*P*-value^*^
Any signs of intracranial hemorrhage—no./total no. (%)	64/345 (18.6)	21/132 (15.9)	0.93
Symptomatic hemorrhage—no./total no. (%)	9/347 (2.6)	1/132 (0.8)	0.30
New ischemic lesions—no./total no. (%)	116/346 (33.5)	39/129 (29.8)	0.45
Space occupying infarctions—no./total no. (%)	33/346 (9.5)	8/131 (6.1)	0.28

* *P-value not adjusted for multiple testing*.

### Treatment Effect of Alteplase According to Smoking Status

**Figure 2:**
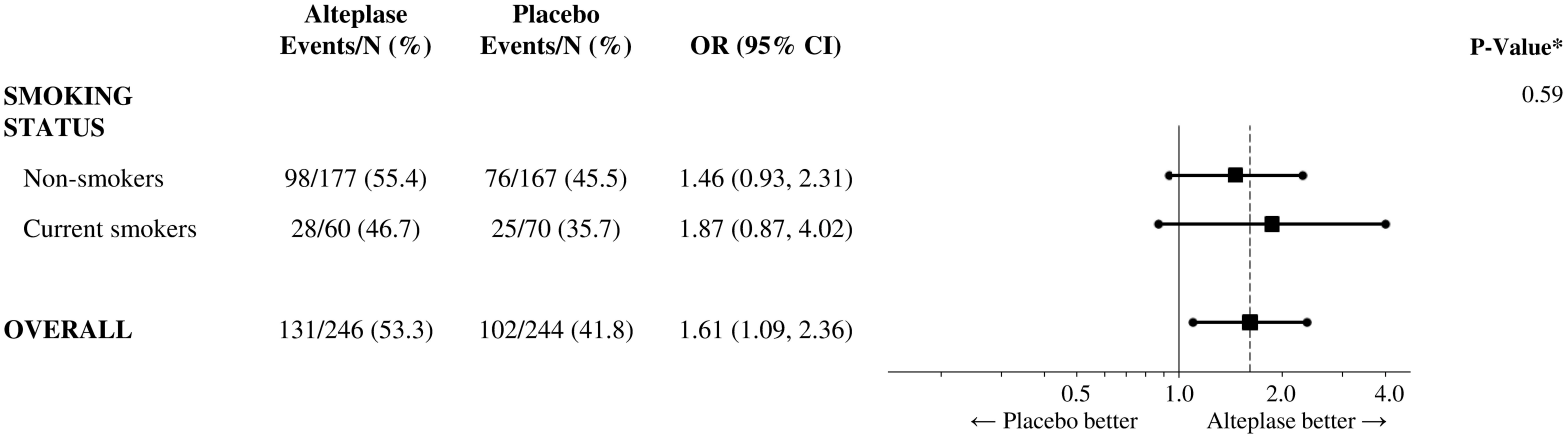
Treatment efficacy of i.v. thrombolysis according to smoking status. Shown are odds ratios for favorable outcome (modified Rankin Scale score 0–1) in current smokers and non-smokers as well as in all participants. *P-value for the test of interaction between treatment group and smoking status. CI stands for confidence interval.

In the total patient sample, multivariate analyses adjusted for age and initial NIHSS score strata showed that treatment with alteplase as compared to placebo was associated with a higher proportion of patients with favorable outcome in dichotomized as well as in mRS shift analysis (OR 1.61 [1.09–2.36], *p* = 0.02; and 1.62 [1.17–2.23], *p* = 0.003, respectively). This treatment effect of alteplase was not different in the group of current smokers and non-smokers (*p*-value for interaction: 0.59 and 0.49, respectively; Figure 2). Similar results were obtained when age (continuous), the presence of proximal large vessel occlusion at baseline and medical history of atrial fibrillation and arterial hypertension were included as additional covariates.

### Exploratory Analyses of Ever-Smokers vs. Never-Smokers

When comparing ever-smokers (combined groups of current smokers and ex-smokers; *n* = 230) with never-smokers (*n* = 256), ever-smokers were significantly younger (mean age 64.2 ± 12.1 vs. 66.5 ± 10.8; *p* = 0.04); more often male (70.3 vs. 57.4%; *p* < 0.01); more often had a history of hypercholesterolemia (43.4 vs. 32.1%, *p* = 0.02) and ischemic stroke (18.4 vs. 8.7%; p < 0.01); and less often had a history of atrial fibrillation (8.3 vs. 16.4%; *p* = 0.02). The remaining baseline characteristics were balanced between the two groups. When adjusting for between group differences at baseline, the rate of favorable functional outcome was similar in both groups (48.9 vs. 47%; *p* = 0.94). The point estimate for the treatment effect of alteplase was higher in the group of ever-smokers than in never-smokers (OR 2.700 [1.520–4.795] vs. 0.913 [0.501–1.665]; *p*-value for interaction = 0.01). The confidence intervals in both groups overlapped and contained the point estimate of the treatment effect of alteplase in the total patient sample (1.61).

## Discussion

We performed a subgroup analysis of the WAKE-UP trial data to investigate the relationship between smoking status and the treatment effect of alteplase. In this study, current smoking did not modify the treatment effect of alteplase in comparison to placebo in patients with AIS with DWI-FLAIR mismatch on MRI.

Patients in the current-smoker group were significantly younger and more often presented with large vessel occlusion, in line with a recent report (14). These associations may be explained by accelerated smoking-induced atheroma formation (15), the pro-coagulatory effects of smoking (16), or different smoking habits in older and younger individuals (17).

The so-called “smoking paradox” refers to the prognostic value of current smoking in patients with AIS treated with alteplase. While some studies have reported an independent beneficial effect of current smoking on functional outcome and mortality (4–6), others have failed to demonstrate such an association after adjustment for confounding covariates (7–9). Data showing an association between current smoking and recanalization of arterial occlusion by means of intravenous thrombolysis (12, 18), intra-arterial thrombolysis (19), or endovascular thrombectomy (20) are more consistent. In contrast to previous studies, data used for the current analysis were collected prospectively and outcome was ascertained in a double-blinded fashion as part of a multicenter randomized controlled trial. All included patients had acute ischemic lesions on MRI. In the population examined for this study, we did not find evidence of better functional outcome in current smokers as compared to non-smokers; if anything, there was a trend for current smokers to have worse functional outcome than non-smokers in adjusted mRS shift analyses. For the first time, we were able to investigate not only the prognostic value of current smoking with regards to clinical outcome, but also its potential to modify the treatment efficacy of alteplase as compared to placebo in patients with MRI-proven AIS, which might be a clinically more relevant parameter that could be used for the individualization of treatment decisions. In line with a previous study that examined the effect of smoking in patients selected on the basis of clinical findings and CT-imaging (10), we did not observe any heterogeneity between current smokers and non-smokers with regards to the treatment effect of alteplase.

Patients planned for endovascular treatment and patients unable to undergo MRI were excluded from WAKE-UP. Therefore, a relatively small proportion of patients had severe stroke symptoms with an NIHSS score > 10. Assuming that active smoking would modify the treatment effect of alteplase preferentially through improved recanalization rates of proximal large vessel occlusion, this association could have been missed in our study due to the small number of patients with proximal large vessel occlusion. Information on recanalization of proximal large vessel occlusions was not available for the current analysis; however, given currently reported recanalization rates of ~20% with alteplase alone (21, 22) event rates in the defined subgroups would have been too low to detect differences between current smokers and non-smokers.

In our analysis, large vessel occlusion was more frequently found in current smokers than in non-smokers. This association has been reported previously by Hendrix et al. (14) in a cohort of 1,654 AIS patients. Evidence by Ntaios et al. (23) indicating that current smoking is predictive of large-artery atherosclerotic stroke etiology rather than microangiopathic stroke combined with the observation that large-artery atherosclerosis represents a major source of large vessel occlusion could explain this association.

Additional exploratory analyses comparing ever-smokers with never-smokers suggested a possible beneficial effect of life-time tobacco exposure on the treatment effect of alteplase. Although the *p*-value for interaction between smoking status (ever-smokers vs. never-smokers) and treatment assignment was statistically significant, the confidence intervals for the treatment effect in the groups of ever-smokers and never-smokers overlapped and both confidence intervals contained the point estimate of the treatment effect in the total patient sample. In addition, the analysis was added *post-hoc* to the analysis plan, which raises the possibility of a type 1 (false positive) error. Nonetheless, lifetime tobacco exposure could lead to vascular alterations of the cerebral circulation such as accelerated formation of arteriosclerosis and atherosclerosis with disturbed endothelial function that may affect the treatment effect of alteplase. Further studies investigating the relationship between ever-smoking vs. never-smoking and the efficacy of thrombolysis are needed. Currently, the results of this exploratory analysis should not affect the decision for or against treatment with intravenous thrombolysis using alteplase in never-smokers.

The following limitations need to be considered when interpreting the findings of our study. First, our study only included patients with AIS and unknown time of symptom onset, mostly nighttime stroke; therefore, our findings may not be generalizable to witnessed strokes occurring during daytime. Second, all patients had to be able to undergo MRI; therefore, our findings may not be generalizable to patients without MRI evaluation. Third, no data was available on the amount or frequency of smoking, nor the specific type of tobacco product utilized. Finally, although patients in our study were randomized to treatment with alteplase or placebo, no randomization was performed with regards to smoking; therefore, even after adjustment for potential confounders in multivariate analyses residual confounding may be present.

## Conclusions

In conclusion, current smokers suffered their strokes at a younger age and more often presented initially with large vessel occlusion and more severe strokes. After adjustment for baseline differences, current smokers had a trend toward worse functional outcome as compared to non-smokers. We found no significant interaction between the treatment effect of alteplase and current smoking status. If the postulated “smoking paradox” does indeed exist, it may be limited to patients with large vessel occlusion. Further studies involving a larger number of more severely affected patients are warranted to investigate this hypothesis.

## Data Availability Statement

The data that support the findings of this study are available from Dr. Thomalla (thomalla@uke.de) upon reasonable request.

## Ethics Statement

The WAKE-UP trial protocol was approved by the national regulatory authority in each of the six participating countries (Belgium, Denmark, France, Germany, Spain, and United Kingdom). The trial was approved by the respective national or local ethics committees or institutional review boards of all participating centers. Patients or their legal representatives provided written informed consent according to national and local regulations. The main trial was conducted according to the principles laid down in the Declaration of Helsinki in its version of Seoul, 2008; the EU Clinical Trial Directive 2001/20/EC; the Note for Guidance on Good Clinical Practice (CPMP/ICH/135/95 of January 17, 1997); the applicable national drug laws, e.g., German Drug Law (Arzneimittelgesetz, 15. Novelle, AMG); and the GCP-Regulation from August 9, 2004.

## Author Contributions

CG and GT conceived and designed the WAKE-UP trial. FB performed the data analysis. SP was part of the central image reading board. LS wrote the first draft of the manuscript. All authors were involved in patient recruitment, interpreted the data, reviewed and edited the manuscript, and approved the final version of the manuscript.

### Conflict of Interest

CG reports receiving lecture fees and advisory board fees from Boehringer Ingelheim, all outside of the submitted work. GT receiving consulting fees from Acandis, grant support and lecture fees from Bayer, lecture fees from Boehringer Ingelheim, Bristol-Myers Squibb/Pfizer, and Daiichi Sankyo, and consulting fees and lecture fees from Stryker, all outside of the submitted work. CS is funded by a grant from Novo Nordisk Foundation and has received lecture fees from Bayer and Boehringer Ingelheim, all outside of the submitted work. KM receiving advisory board fees and drugs supplied by Boehringer Ingelheim, and advisory board fees from Bayer and Daiichi-Sankyo, all outside of the submitted work. SP receiving fees for serving on an imaging board from Lundbeck, all outside of the submitted work. MEn receiving fees for serving as a principal investigator, fees for serving on a steering committee, lecture fees, consulting fees, and advisory board fees, paid to his institution, and grant support from Bayer, lecture fees and advisory board fees, paid to his institution, from Boehringer Ingelheim and Amgen, lecture fees, paid to his institution, from Bristol-Myers Squibb/Pfizer, GlaxoSmithKline, Sanofi, Ever Pharma, and Novartis, and advisory board fees, paid to his institution, from Daiichi Sankyo and Covidien, all outside of the submitted work. The remaining authors declare that the research was conducted in the absence of any commercial or financial relationships that could be construed as a potential conflict of interest.

## Abbreviations

AIS: acute ischemic stroke
DWI: diffusion-weighted imaging
FLAIR: fluid attenuated inversion recovery
IQR: interquartile range
MRI: magnetic resonance imaging
mRS: modified Rankin scale
NIHSS: National Institutes of Health Stroke Scale (score)
OR: odds ratio
SD: standard deviation.
